# Clinical Characteristics, Comorbidities, and Outcome of Critically Sick Patients With COVID-19 Pneumonia Admitted in the Intensive Care Unit of a Tertiary Care Hospital in Lahore, Pakistan: A Retrospective Cohort Study

**DOI:** 10.7759/cureus.25286

**Published:** 2022-05-24

**Authors:** Aijaz Zeeshan Khan Chachar, Mohsin Asif, Khushbakht Tanveer, Muhammad K Rauf, Javeid Iqbal, Kinza Khan, Zohaib Khan, Bilal Bin Younis, Khurshid Khan

**Affiliations:** 1 Medicine, Fatima Memorial Hospital College of Medicine and Dentistry, Lahore, PAK; 2 Medicine, Fatima Memorial Hospital, Lahore, PAK; 3 Medicine, The University of Tennessee Health Science Center, Memphis, USA; 4 Medicine, Shaikh Zayed Hospital, Lahore, PAK; 5 Medicine and Endocrinology, Fatima Memorial Hospital College of Medicine and Dentistry, Lahore, PAK

**Keywords:** ischemic heart disease, hypertension and covid-19, covid-19 and diabetes, covid-19 pneumonia, intensive care, critically sick, covid-19, comorbidities

## Abstract

Background

Coronavirus disease 2019 (COVID-19), caused by severe acute respiratory syndrome coronavirus 2 (SARS-CoV-2), is a multisystem disease that primarily involves the respiratory tract. The first case of COVID-19 was identified in late 2019 in the province of Wuhan, China, which was followed by the rapid spread of the disease globally, becoming a present-day pandemic.

Objectives

The aim of this study is to describe the clinical characteristics, comorbidities, and outcomes of critically sick patients with COVID-19 pneumonia admitted to the intensive care unit (ICU) of Fatima Memorial Hospital, Lahore, from March 2021 to August 2021. A total of 133 patients were chosen for this retrospective cohort study.

Results

There was a total of 133 patients, out of which 65 (48.9%) were male and 68 (51.1%) were female. Of these 133 patients, 70 (52.6%) were discharged home after recovery and 63 (47.4%) died; 96 (72.2%) patients had diabetes mellitus and of these, 53 (55.2%) patients died and 43 (44.8%) were discharged, 94 (70.7%) patients had hypertension, out of which 53 (56.4%) died and 41 (43.6%) were discharged home, 40 (30.1%) patients had ischemic heart disease (IHD), out of which 28 (70%) died and 12 (30%) were discharged. A total of 48 (36.1%) patients needed invasive positive pressure ventilation (IPPV) and 78 (58.6%) patients required noninvasive positive pressure ventilation (NIPPV).

Conclusion

Patients with one or more underlying co-morbidities had poor clinical outcomes compared to those with no co-morbidities, with the most vulnerable group being patients with Ischemic heart disease, chronic kidney disease, hypertension, and diabetes mellitus in descending order.

## Introduction

Coronavirus disease (COVID-19), caused by severe acute respiratory syndrome coronavirus 2 (SARS-CoV-2), is a multisystem disease that primarily involves the respiratory tract. The first case of COVID-19 was identified in late 2019 in the province of Wuhan (Hubei) in China, which was followed by the rapid global spread of the disease. The outbreak of COVID-19 was labeled as a pandemic by the World Health Organization (WHO) on March 11, 2020 [1].

The first case of COVID-19 in Pakistan was reported on February 26, 2020, in Karachi [2], and by May 2022, there were around 1.5 million confirmed cases of COVID-19 in the country with around 30,300 deaths as per the WHO [3].

COVID-19 presents with a varying degree of severity, ranging from mild upper respiratory tract infection to multi-organ failure and it has been estimated that around 5-20% of the patients develop critical illness requiring intensive care unit (ICU) care [4].

At this point, there are a limited number of studies conducted in tertiary care hospitals in Pakistan to analyze the clinical course and outcomes of critically sick COVID-19 patients admitted to the ICU. Therefore, we conducted this study to describe the demographics, clinical characteristics, and outcomes of the patients with COVID-19 admitted to the ICU of Fatima Memorial Hospital (FMH), which is a tertiary care hospital in the city of Lahore, Pakistan.

## Materials and methods

Objectives/outcomes

The primary endpoints were to determine the average length of stay, ICU mortality, and risk factors associated with poor outcomes in critically sick patients with COVID-19 pneumonia admitted to the ICU. The secondary endpoint of this study was to describe the clinical and laboratory parameters of critically sick patients admitted with COVID-19 pneumonia in ICU at the time of initial presentation.

Operational definition

Patients were labeled with COVID-19 pneumonia based on typical clinical features of COVID-19 pneumonia and typical radiological findings suggestive of COVID-19 pneumonia with or without a positive COVID-19 polymerase chain reaction (PCR) test.

Methodology

This was a single-center, non-interventional, retrospective cohort study of critically sick patients with COVID-19 pneumonia admitted to the ICU of Fatima Memorial Hospital, a 470 bedded tertiary care teaching hospital located in Lahore, Pakistan, from March 2021 to August 2021. The records of 133 patients admitted to the ICU of the hospital were studied.

Critically ill patients above 18 years of age who were admitted to COVID-19 ICU with a diagnosis of COVID-19 pneumonia based on a positive reverse transcriptase PCR for COVID-19 or high-resolution computed tomography (HRCT) chest findings suggestive of COVID-19-related lung injury were included in the study.

Data related to their epidemiological, demographic, clinical, comorbidities, and outcomes were collected and analyzed. This included the Sequential Organ Failure Assessment (SOFA) score, oxygen requirement, C-reactive protein (CRP), lactate dehydrogenase (LDH), ferritin, procalcitonin, and d-dimer at the time of admission. All the data were entered on a structured proforma and analyzed using IBM SPSS Statistics for Windows, Version 25.0 (Released 2017; IBM Corp., Armonk, New York, United States). Frequency and percentages were calculated for the qualitative variables such as gender, comorbidity, symptoms, and outcomes. Quantitative variables of the study, such as SOFA, CRP, LDH, procalcitonin, D-dimers, and length of ICU stay, were expressed as median (interquartile range (IQR)). A Chi-square test was applied, as appropriate.

Ethical considerations

Patients were included in the study after approval by the Institutional Review Board, Fatima Memorial Hospital College of Medicine & Dentistry, Lahore, Pakistan (approval number FMH-27/12/2021-IRB#983).

## Results

Of the total of 133 patients, 65 (48.9%) were male and 68 (51.1%) were female; 23 patients (17.3%) were less than the age of 50 years, 36 patients (27.1%) were older than 70 years, and 74 patients (55.6%) were between the ages of 50 and 70 years. Overall, there was a much higher patient population in the older age groups (Table 1). The median oxygen requirement was 10 liters with an IQR of 6 liters. The median CRP was 90 with an IQR of 88. The median SOFA score was 4 with an IQR of 2. The median procalcitonin was 24 with an IQR of 26.7. In our cohort, 48 (36.1%) patients required invasive positive pressure ventilation (IPPV) and 78 (58.6%) required noninvasive positive pressure ventilation (NIPPV) (Table 1).

**Table 1:** Clinical characteristics, laboratory parameters, and comorbidities IQR: interquartile range; CRP: C-reactive protein; SOFA: Sequential Organ Failure Assessment; NIPPV: non-invasive positive pressure ventilation; IPPV: invasive positive pressure ventilation

Variables	N (%)
Age categories	
< 50 years	23 (17.3)
50-70 years	74 (55.6)
>70 years	36 (27.1)
Gender	N (%)
Male	65 (48.9)
Female	68 (51.1)
Median Initial laboratory measures (IQR)	
Respiratory Rate (IQR)	80 (20)
Oxygen Requirement at Presentation (IQR)	10 (6)
CRP (IQR)	90 (88)
SOFA Score (IQR)	4 (2)
Procalcitonin (IQR)	24 (26.7)
Comorbidities	N (%)
Diabetes Mellitus	96 (72.2)
Hypertension	94 (70.7)
Chronic Kidney Disease	12 (9)
Ischemic Heart Disease	40 (30.1)
Need for NIPPV	78 (58)
Need for IPPV	48 (36.1)

The average total length of stay in discharged patients was eight days with an IQR of three days and in non-survivor patients was 11 days with an IQR of two days. Out of the 133 patients, 70 (52.6%) patients were discharged and sent home and 63 (47.4%) patients expired in ICU. According to age, of the 63 expired patients, 7 (11.1%) were below 50 years of age, 30 (47.6%) were between 50-70 years of age and 26 (41.3%) patients were above 70 years of age (Table 2). Regarding gender and mortality, 29 (46%) of the 63 expired patients were male and 34 (54%) were female (Table 2). Out of the total 133 patients, 96 (72.2%) had diabetes mellitus and of the diabetic patients, 53 (55.2.1%) died and 43 (44.8%) were discharged (Table 2). Of the total of 133 patients, 94 (70.7%) had hypertension, out of which, 53 (56.4%) died and 41 (43.6%) were discharged home (Table 2). Of the 40 (30.1%) patients who had ischemic heart disease, 28 (70%) died and 12 (30%) were discharged and of the 12 (9%) patients who had chronic kidney disease, seven (58.33%) died and five (41.7%) were sent home (Table 2).

**Table 2:** Outcomes of patients with regard to age, gender, comorbidities, and need for NIPPV/IPPV DM; Diabetes mellitus, HTN; Hypertension, CKD; Chronic kidney disease; IHD; Ischemic heart disease; IQR, interquartile range; NIPPV: non-invasive positive pressure ventilation; IPPV: invasive positive pressure ventilation

Variables	Discharged N (%)	Expired N (%)
Age categories		
< 50 years	16 (22.9)	7 (11.1)
50- 70 years	44 (62.9)	30 (47.6)
>70 years	10 (14.3)	26 (41.3)
Gender		
Male	36 (51.4)	29 (46)
Female	34 (48.6)	34 (54)
Comorbidities		
DM	43 (44.8)	53 (55.2)
HTN	41 (43.6)	53 (56.4)
CKD	5 (41.7)	7 (58.33)
IHD	12 (30)	28 (70)
Patients on IPPV	0 (0)	48 (100)
Patients on NIPPV	15 (19.3)	63 (80.7)
Length of Stay in ICU (IQR)	8 Days (3)	11 Days (2)

## Discussion

This is one of few studies from Pakistan demonstrating the impact of clinical variables on the outcome of critically sick COVID-19 pneumonia patients admitted to ICU.

In our study, the majority of the patients had multiple comorbid conditions. A retrospective cohort study of a similar design was conducted at King Abdul Aziz University Hospital, one of the major tertiary care hospitals in the Kingdom of Saudi Arabia, by Al Sulaiman et al. [5]. In that study, 40% of the participants were above 65 years of age. Comparatively, in our study, 27.1% of patients were more than 70 years of age and 55.6% were between the ages of 50-70 years. So, there were slightly higher numbers of patients in our study in older age brackets. In the study of Al Sulaiman et al. [5], the survival rate was 52.6% while our study showed a survival rate of 53%. The mortality data in both studies is quite comparable.

In a similar study conducted in New Jersey, United States, by Cedano et al. [6], out of 132 patients admitted to the ICU, 45% had diabetes mellitus and 59% had hypertension. In our study, 72.2% were diabetics and 70.7% were hypertensive. Comorbidity data in both studies were quite comparable. The mortality rate in the study conducted by Cedano et al. [6], the mortality rate was 69% while that in our study was 47%. This somewhat better outcome in our study can be attributed to the fact that our data were from the third COVID-19 wave while Cedano et al.'s study data were from the first wave. Improved treatment protocols in the third wave might be the reason for the difference in mortality between the two studies, with resultant better outcome in our study.

A study conducted at two hospitals affiliated with Columbia University Irving Medical Center, New York, United States, by Cummings et al. [4] revealed that most of the mortalities were in the age group greater than 50 years. In their study, 78% of patients were above 50 years of age and in our study, 82.7% were above 50 years. In our study, 58% of patients required NIPPV and 36.1% required IPPV, while in the study by Cummings et al. [4], 62% of patients needed NIPPV and 41% required IPPV, which is highly comparable. In the study by Cummings and colleagues [4], mortality was 39.2% compared with 47.4% in our study. The difference can be explained by the fact that the study by Cummings et al. included COVID-19 patients admitted in both the ICU and the High Dependency Unit (HDU), whereas our study included very critical patients only in the ICU.

Filardo et al. [7] conducted a similar study at Bellevue Hospital, New York, United States, in which 23% of patients were aged less than 50 years and 31.5% were aged more than 65 years. In our study, 17.3% were younger than 50 years and 82.7% were older than 50 years. Regarding comorbidities, in the study by Filardo et al., 33% of patients had diabetes mellitus, chronic kidney disease was seen in 7% of patients and ischemic heart disease was present in 51.5% [7]. In comparison, in our study, 72.2% of patients had diabetes mellitus, 9% had chronic kidney disease, and 30.1% had ischemic heart disease. In our study, we had a much higher patient population with diabetes. The median length of ICU stay was eight days in our study and six days in the study conducted by Filardo et al. [7]. Out of 135 ICU patients in their study, 53% expired [7]. The mortality was significantly lower in our study (47.4%), especially considering that our cohort had a much higher number of diabetic patients.

Limitations

This was a single-center study and the sample size was small; However, the novelty of the disease outweighed the sample size. Also, it was conducted in one city in Pakistan and, therefore, results cannot be generalized to the entire country. More studies from different parts of the country are needed with the collaboration of experts dealing with this novel disease for a better understanding of this devastating disease.

## Conclusions

Patients with one or more underlying comorbidities had poor clinical outcomes compared to those with no comorbidities, with the most vulnerable group in terms of mortality being the patients with ischemic heart disease (70%), chronic kidney disease (58.33%), hypertension (56.4%), and diabetes mellitus (55.2%) in descending order.

## References

[1] (2020). -World Health Organization. Coronavirus disease 2019 (COVID-19) situation report-51. Geneva, Switzerland: World Health Organization. Coronavirus Disease 2019 (COVID-19): Situation Report - 51.

[2] Abid K, Bari YA, Younas M, Tahir Javaid S, Imran A (2020). Progress of COVID-19 epidemic in Pakistan. Asia Pac J Public Health.

[3] (2021). World Health Organization: Pakistan. https://www.who.int/countries/pak.

[4] Cummings MJ, Baldwin MR, Abrams D (2020). Epidemiology, clinical course, and outcomes of critically ill adults with COVID-19 in New York City: a prospective cohort study. Lancet.

[5] Al Sulaiman KA, Aljuhani O, Eljaaly K (2021). Clinical features and outcomes of critically ill patients with coronavirus disease 2019 (COVID-19): A multicenter cohort study. Int J Infect Dis.

[6] Cedano J, Fabian Corona E, Gonzalez-Lara M (2021). Characteristics and outcomes of patients with COVID-19 in an intensive care unit of a community hospital; retrospective cohort study. J Community Hosp Intern Med Perspect.

[7] Filardo TD, Khan MR, Krawczyk N (2020). Comorbidity and clinical factors associated with COVID-19 critical illness and mortality at a large public hospital in New York City in the early phase of the pandemic (March-April 2020). PLoS One.

